# The tentative application of en bloc concept in the pediatric brain tumor: Experience from a large pediatric center in china

**DOI:** 10.3389/fonc.2022.1018380

**Published:** 2022-11-11

**Authors:** Liangliang Cao, Shuaiwei Tian, Wenkun Ma, Zhouwen Ni, Gang Tian, Yang Zhao, Qinhua Wang, Zhen Xu, Jiajia Wang, Zhuangzhuang Liang, Heng Zhao, Lingrui Yang, Baocheng Wang, Jie Ma

**Affiliations:** ^1^ Department of Pediatric Neurosurgery, Xinhua Hospital Affiliated to Shanghai Jiao Tong University School of Medicine, Shanghai, China; ^2^ Department of Epidemiology and Medical Statistics, Xiangya School of Public Health, Central South University, Changsha, Hunan, China

**Keywords:** en bloc, piecemeal, pediatric, brain tumor, gross total resection

## Abstract

**Background:**

Children are more susceptible to the higher rate of massive blood transfusion because of the less allowable blood loss and lower intraoperative tolerance to blood loss during the resection of brain tumors. The surgical concept of en bloc resection, which is widely used in other tumors, may contribute to the improvement of brain tumor resection. However, there is still a lack of comprehensive research on its application in pediatric brain tumors.

**Objective:**

The aim of this study is to investigate the outcomes of the en bloc concept and the factors associated with the application of the en bloc concept in pediatric brain tumors.

**Methods:**

According to the surgical concept involved, the patients were divided into three subgroups: complete en bloc concept, partial en bloc concept, and piecemeal concept. The matching comparison (complete and partial en bloc concept groups vs. piecemeal concept group) was conducted to investigate the effect of the en bloc concept on the outcomes. Then, the patient data from January 2018, when the en bloc concept was routinely integrated into the brain tumor surgery in our medical center, were reviewed and analyzed to find out the predictors associated with the application of en bloc concept.

**Results:**

In the en bloc group, the perioperative parameters, such as hospital stay (p = 0.001), pediatric intensive care unit (PICU) stay (p = 0.003), total blood loss (p = 0.015), transfusion rate (p = 0.005), and complication rate (p = 0.039), were all significantly improved. The multinomial logistic regression analysis showed that tumor volume, bottom vessel, and imaging features, such as encasing nerve or pass-by vessel, finger-like attachment, ratio of “limited line”, and ratio of “clear line”, were independent predictors for the application of the en bloc concept in our medical center.

**Conclusion:**

This study supports the application of complete and partial en bloc concept in the pediatric brain tumor surgery based on the preoperative evaluation of imaging features, and compared with the piecemeal concept, the en bloc concept can improve the short outcomes without significant increases in the neurological complications. Large-series and additional supportive pieces of evidence are still warranted.

## Introduction

In the daily practice of brain tumors surgery, neurosurgeons always have to be faced with massive intraoperative hemorrhage. Different from the relative larger allowable blood loss and compensative capacity of adult patients, pediatric cases, especially neonates, have a smaller blood volume and a lower tolerance to intraoperative blood loss and, thus, a higher rate of massive blood transfusion that is often associated with the high mortality (1–3). Despite the more efficient treatment regimens differing markedly from the single-modality therapy to combinations of surgery, systemic therapy, and targeted agents, the brain tumor keeps the leading cause of morbidity and mortality in pediatric cancers all along (4–6). In consequence, it is sensible to improve the surgical concept and operation skills.

**Figure 1:**
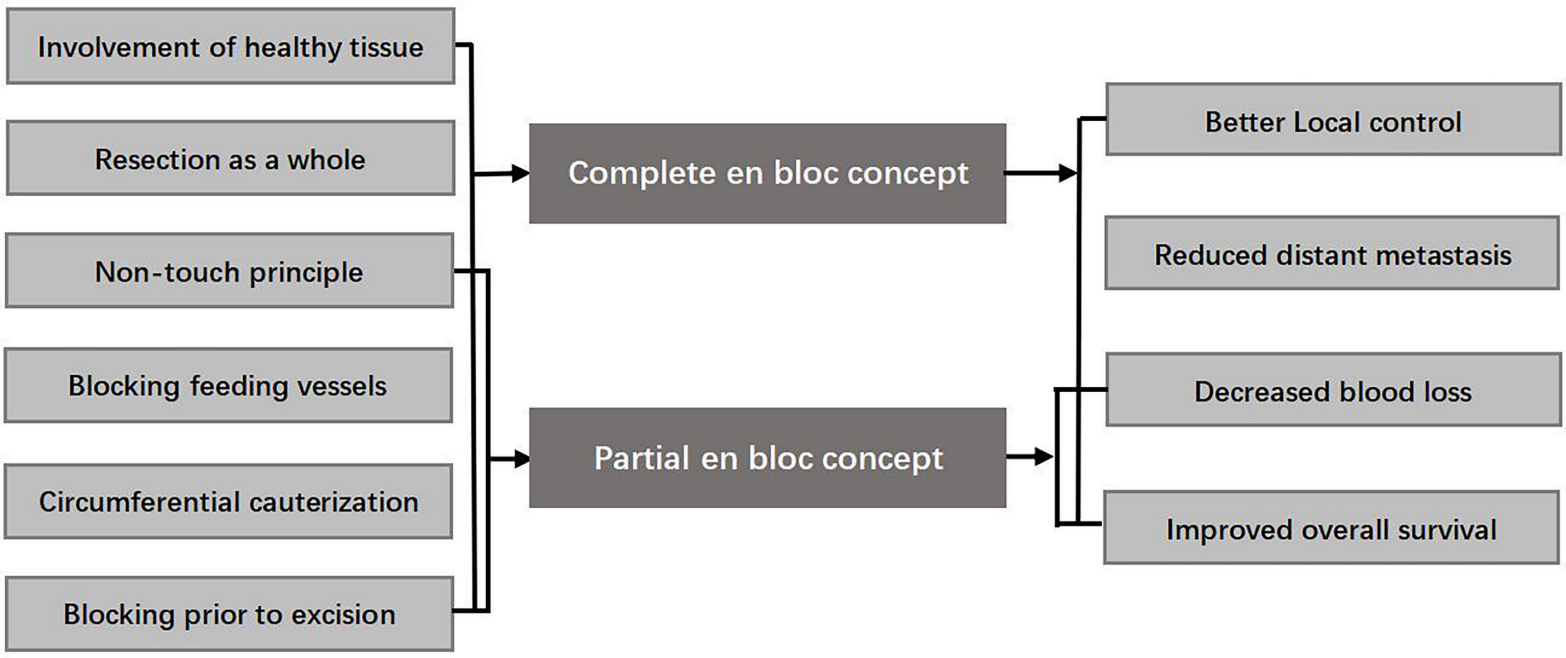
Complete and partial en bloc concept involved in this study and their hypothetical effects.

Considering the application of en bloc technique in oncology and scattered reports in brain tumor, it is possible to be realized in selective cases (7–13). Furthermore, part of surgical concept-circumferential cauterization and isolation involved in the en bloc concept can block the feeding arteries and may help to decrease blood loss. Therefore, combining the respective advantages of piecemeal and en bloc concept involved in previous studies (**Figure 1**) (7, 8, 14–18), we introduced the complete or partial en bloc concept into the surgery. In this study, we investigated the effect of the en bloc concept on the short outcome and the factors associated with its application in the pediatric brain tumors. Furthermore, this is also the first comprehensive report about application of the en bloc concept in the brain tumors.

## Materials and methods

### Study design

**Figure 2:**
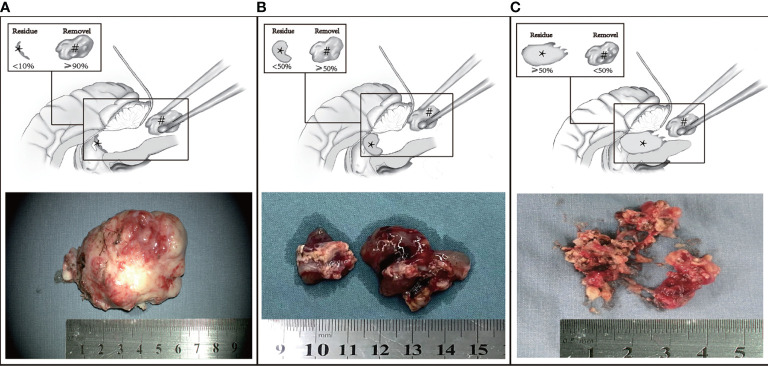
Three methods of resection. **(A)** Complete en bloc concept (marginal resection as a whole ≥90% and piecemeal resecting <10%). **(B)** Partial en bloc concept (marginal resection ≥50% and <90%). **(C)** Piecemeal concept (piecemeal resecting >50%). The black asterisk and black pound sign represent residual tumor and the part removed as a block.

**Figure 3:**
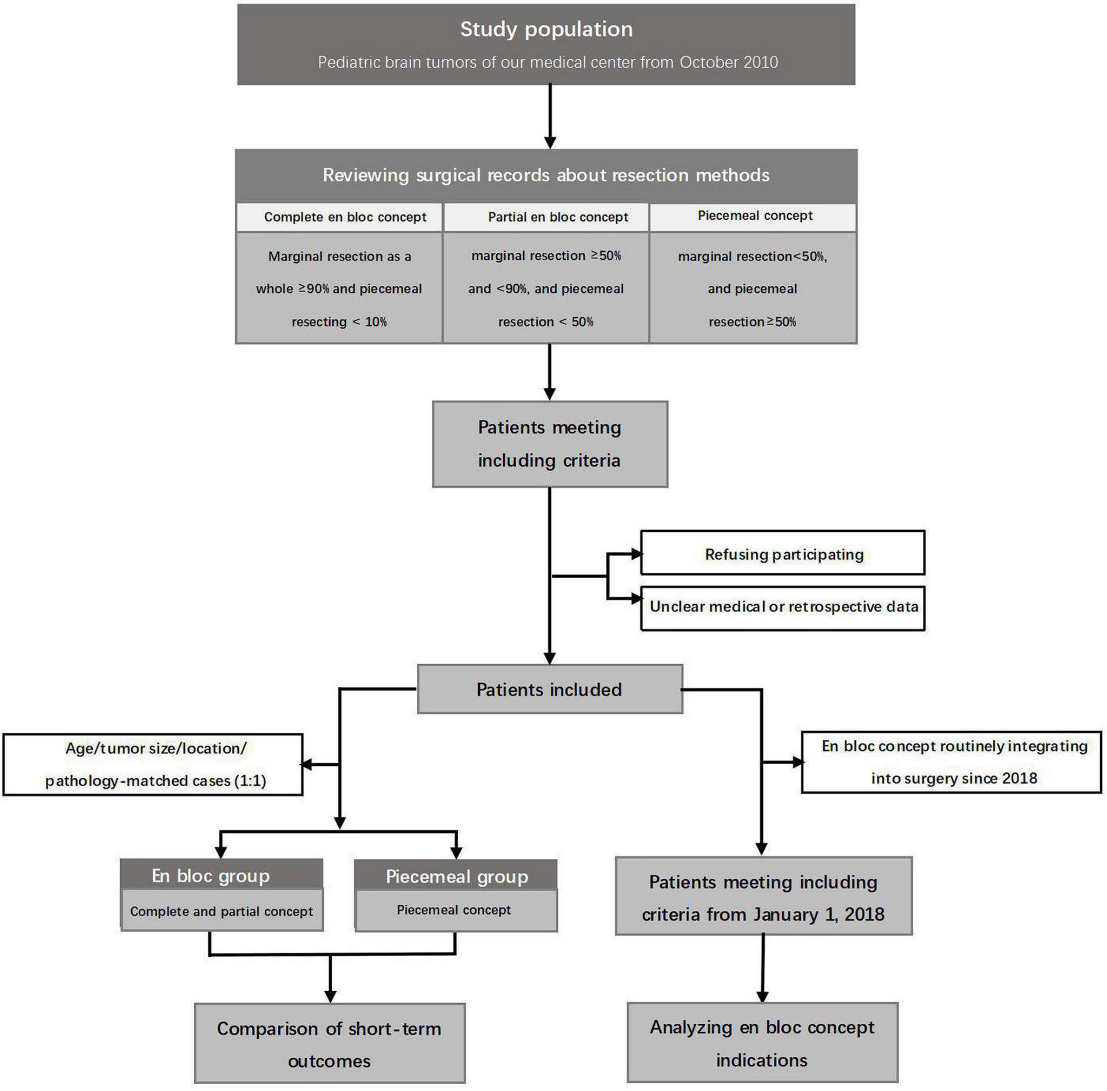
The flow chart of this study.

In this retrospective study, all the medical data were prospectively collected from our medical center, which was one of the major pediatric medical centers in China. To illustrate the application of the en bloc concept in brain tumors, three methods were defined as complete en bloc resection (**Supplemental Digital Content 1**), partial en bloc resection (**Supplemental Digital Content 2**), and piecemeal resection (**Figure 2**). To investigate the outcome and the effect of the en bloc concept on the control of perioperative complication, the matching comparison (complete and partial en bloc concept groups vs. piecemeal concept group) was conducted on the basis of age (± 2 years), lesion size (± 3cm^3^), tumor location, and neoplasia pathology, at a ratio of 1:1. Considering the fact that intraoperative navigation was not applied in all patients, the time of operation was normalized by removing the time of navigation. In our medical center, the en bloc concept has been routinely considered with priority since January 2018, according to the intraoperative tumor characteristics. To investigate the factors associated with the application of en bloc concept, the medical records of this patient set were reviewed and analyzed (**Figure 3**). All the operation consents were obtained during hospitalization. Informed consents of this study were obtained by mails, and Institutional Review Board approval was obtained.

### Patient selection

The inclusion criteria were as follows: 1) primary isolated brain tumors and experienced surgical treatment by single senior surgeon in our medical center; 2) clear medical or retrospective data needed; 3) first intracranial surgery; 4) performed by the same neurosurgeon; 5) patients included in the matching comparison had at least 2 years of follow-up. The exclusion criteria were as follows: 1) intraoperative diffused tumor seeding and/or the evidence of positive CSF cultures like leptomeningeal spread; 2) radiologic evidence of metastasis from the brain tumors; 3) multiple primary brain tumors with the same pathological features; and 4) biopsy only.

### Ascertainment of covariates

**Figure 4:**
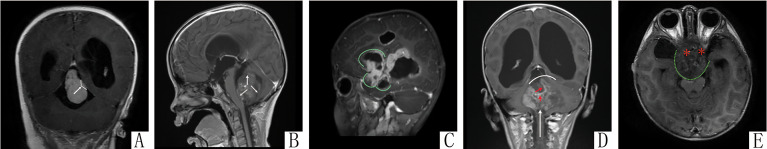
The imaging features related to the en bloc concept application. **(A)** The “limited line”–transverse T1 MRI confirming the boundary line (black arrow) between tumor (block pound sign) and the right thalamus (black asterisk). **(B)** The “clear line”–sagittal T1 post-contrast shows the thin hypointense gap between tumor and brainstem, velum medullare superius, and uvula of vermis (white arrow); **(C)** The “finger-like” attachment-Sagittal T2 post-contrasting MRI showing the “Cauliflower-like” outline of tumor. **(D)** The bottom vessel–sagittal T1 post-contrast MRI showing a feeding vessel (red arrow) originating from the “bottom” (white curve) of tumor that is relative to the direction of surgical approach (white arrow). **(E)** The “perforating nerves or vessels”–sagittal T1 post-contrast MRI showing the affected auditory and cranial nerve (red asterisk).

In addition to the pertinent variables prospectively recorded in the electronic medical records (as shown in **Table 1**), the tumor characteristics, like cystic or solid and tumor depth (“Deep” location was defined as any subcortical location below the deepest adjacent sulcus in close proximity to the basal ganglia and/or thalamus, and vice versa as “superficial”) (19), and radiologic features were collected from operation notes and preoperative imaging data. Because of the uneven tumor size in different regions, the ratio of “limited line”–maximal length of tumor boundary adjacent to critical structures to the circumference of tumor margin in the corresponding sagittal, coronal, and axial planes on preoperative MRI was classified as three levels by 10% and 50% (**Figure 4A**). Likewise, the ratio of “clear line”–maximal length of well-defined or hyperintense boundary between the tumor lesion and surrounding parenchyma on contrasted T1-MRI was graded into three levels by 50% and 90% (**Figure 4B**). The “finger-like” boundary meant intended tumor margin (**Figure 4C**). The “bottom vessel” meant any kind of identifiable vessels on MRI feeding from or passing through the tumor bottom relative to direction of surgical approach (**Figure 4D**). Whether the tumor encased critical neurovascular structures or pass-by vessels was also recorded (**Figure 4E**).

**Table 1:** Short outcomes between the en bloc group and the piecemeal group.

	En bloc group	Piecemeal group	*P-*value
**Mean age (SD, years)**	4.60 (3.55)	4.47 (3.30)	0.227
**Sex (male/female)**	31/25	30/26	0.283
**Hospital stay (SD, days)**	19.29 (3.63)	24 (3,910.46)	**0.001**
**PICU (SD, days)**	2.29 (0.87)	3.43 (2.46)	**0.003**
**Tumor volume (SD, cm^3^)**	30.66 (20.64)	30.00 (20.75)	0.382
**Duration of surgery (SD, min)**	218.30 (34.76)	293.66 (80.39)	**0.000**
**Duration of resection (SD, min)**	45.59 (15.01)	86.47 (23.82)	**<0.001**
**Total blood loss (SD, ml)**	408.51 (177.93)	506.41 (226.43)	**0.015**
**Blood loss of resection (SD, ml)**	215.85 (135.62)	340.63 (201.35)	**<0.01**
**Transfusion rate**	30 (53.57%)	44 (78.57%)	**0.005**
**EOR**			
Gross total	47 (83.93%)	39 (69.64%)	0.046
Subtotal	7	10	
Partial	2	7	
**Overall complications**	30 (53.57%)	40 (71.43%)	**0.039**
**Major systemic complications**	10 (17.86%)	14 (25%)	0.245
**Major neurological complications**	2 (3.57%)	6 (10.71)	0.135
**Minor complications**	18 (25%)	28 (39.29)	**0.042**
**Postoperative headache**	9 (16.07%)	16 (28.57%)	0.086
**Fever**	21 (37.5%)	33 (57.89%)	**0.019**
**VPS**	3 (5.36%)	7 (12.5%)	0.160
**Recurrence rate**	8 (14.29%)	12 (21.43%)	0.230
**Metastatic rate**	3 (5.36)	5 (8.93%)	0.358
**Perioperative mortality**	2 (3.57%)	6 (10.71%)	0.135
**Survival rate**	48 (85.71%)	47 (83.93%)	0.500

The meaning of the bold values means the results is statistically signaficant.

### Surgical procedure

**Figure 5:**
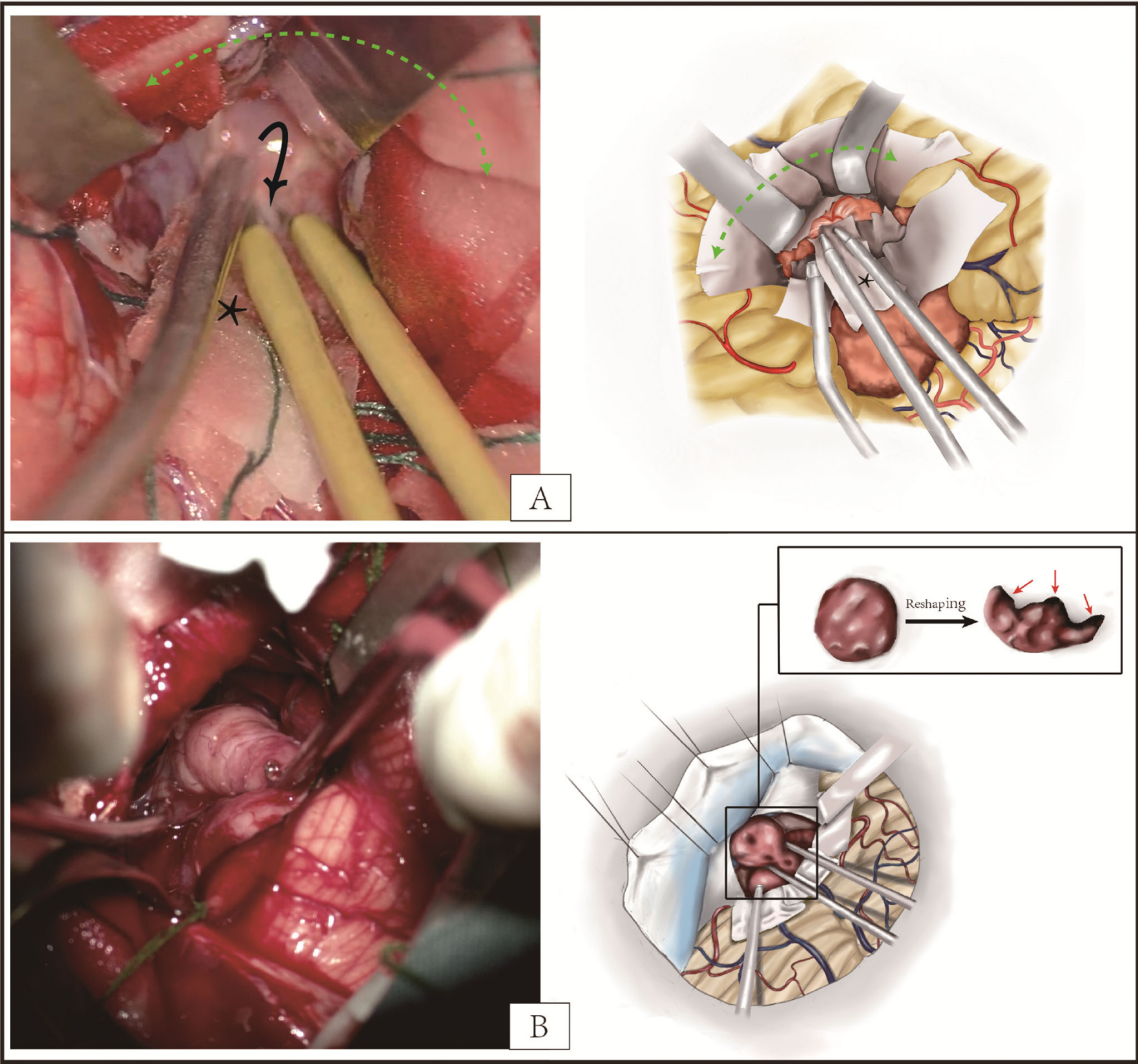
**(A)** The exposure of ependymoma in the posterior fossa. The “tumor-side” manipulation (black arrow) can avoid the excessive stretch on adjacent parenchyma when exposing the deep feeding vessels. The cottonoids strip (black asterisk) can serve as a screen between tumor and bipolar coagulation to reduce superficial contact hemorrhage and tumor implantation mediated by iatrogenic operation. According to the “non-touching” principle from en bloc concept, more cottonoids strips (blue asterisk) are advocated for reduction of tumor seeding in addition to protection of cerebellum. **(B)** The reshaping of tumor by bipolar coagulation for deep exposure or extraction resulting from limited incision. Different from the piecemeal resection or reshaping by micro-forceps, reshaping by bipolar coagulation can maximal the integrity of lesion to reduce the tumor cell spreading and hyperthermia inactivation of cut section (red arrow), which comes from the “taking as a whole” principle of en bloc concept.

On the basis of the suggestions of neuro-oncology multidisciplinary team, the meticulous choice between en bloc and piecemeal resection was made for each case enrolled according to their preoperative data like the reconstructed tumor model and digital substraction angiography (DSA) (see **Supplemental Digital Content 3**). Radiologist helped to identify the functional areas, such as eloquent brain regions and basal ganglia. DSA was able to provide the detailed information of supplying vessels of tumor with abundant blood supply, whereas the tumor model was able to provide the optimal choice of surgery approach and protocol to reach the supplying vessels. Surgical approaches were planned routinely according to the tumor locations. The optimal incision and approach to the lesion and target vascular pedicles could be obtained if the neurosurgical navigation systems (NNS, StealthStation S7, Medtronic, USA) was available. For the invasive lesions with peripheral hyperintense stripe on T2-MRI or clear boundary on T1 contrast-enhanced MRI, NNS and intraoperative neurophysiological monitoring were indispensable for the clear identification of “radiological margin” (see **Supplemental Digital Content 4**) and feeding vessels (see **Supplemental Digital Content 5**). In addition, during the marginal resection, the “tumor-side” manipulation (**Figure 5A**) or draining part of cyst fluid could work to avoid the extra squeeze effect on the adjacent parenchyma. When the cortex incision or surgical passage limited giant lesions being taken out as a whole, reshaping them according to the surgical corridor by bipolar coagulation after the tumor isolation was also a viable alternative (**Figure 5B**).

### Statistical analysis

Statistical analyses were performed using SPSS (version 22.0, IBM Corp). The pair-sample test, Wilcoxon signed-rank test, and McNemar test were performed, respectively, for paired normally distributed, skewed distributed, and categorical data. The statistical comparisons among the three methods (compete en bloc, partial en bloc, and piecemeal) were analyzed with one-way ANOVAs with Bonferroni correction for normally distributed values, Kruskal–Wallis tests for skewed distribution, and Fisher exact test for categorical data. In addition, univariate and multivariate multinomial logistic regression analyses were used to identify variables associated with application of complete or partial en bloc concept. Factors with a p < 0.1 were introduced for cumulative odds logit models building. All analyses were performed using a two-sided type 1 error rate of 0.05 as the threshold for statistical significance.

## Results

### Comparison of short outcomes

As shown in **Table 1**, the matched comparison was based on the data of 112 patients with comparable demographic data (ages: 4.60 ± 3.55 vs. 4.47 ± 3.30, p = 0.227) and tumor characteristics. In addition, significant differences were observed in perioperative parameters, such as hospital stay (p = 0.001), PICU stay (p = 0.003), duration of resection (p = 0.000), total blood loss (p = 0.015), blood loss of resection (p = 0.001), and transfusion rate (p = 0.005), and in the en bloc group, they were all significantly improved. The gross total resection (GTR) rate in the en bloc group was significantly improved from near 70% to over 80% (p = 0.046). When comparing the complication rates, all kinds of complication rates were lower than that in the piecemeal group, although with only marginal difference in overall complication rate (P = 0.039), minor complication rate (P = 0.042), and postoperative fever (P = 0.019). There was no significant difference in 2-year survival rate, recurrence rate, and metastatic rate between the two groups, but they were all lower numerically in the en bloc group.

### Factors associated with the application of en bloc concept

A total of 171 cases, from 1 January 2018, owned the complete medical data and clear retrospective data and agreed to participate in this study. Most (about 53%) of them experienced partial en bloc concept resection (mean age: 4.86 ± 3.51 years), followed by the complete en bloc resection (28.65%), and only less than 20% cases underwent conventional piecemeal resection. The mean operation time of the complete en bloc group was significantly lower than that of the other two groups (P < 0.05), and total blood loss was also the least. The top three tumor types were medulloblastoma, ependymoma, and pilocytic astrocytoma, and the majority of medulloblastomas were removed by partial en bloc conception. According to the WHO classification, the en bloc concept was allied mostly in the high-grade lesions (86.67%, complete or partial). Except for the sellar tumors, over 70% of lesions were removed by complete or partial en bloc conception in the other intracranial regions. Apart from that, tumor characteristics, growth pattern, and imaging features were significantly different among the three groups (**Table 1**). In the univariate analysis, patients’ age, tumor type, tumor location, tumor volume, bottom vessel, encasing nerve or pass-by vessel, finger-like attachment, ratio of “limited line”, and ratio of “clear line” were found to be significant different among the three groups of tumors (P < 0.05) (**Table 2**).

**Table 2:** Demographic and tumor characteristics.

Variable		Surgical group			
	Overall	Complete en bloc	Partial en bloc	Piecemeal resection	*P-*value
**No. of patients**	171	49 (28.65%)	91 (53.22%)	31 (18.13%)	
**Mean age (SD, years)**	4.86 (3.51)	5.96 (3.51)	4.61 (3.42)	3.86 (3.44)	**0.016**
**Males**	96 (56.14%)	30	52	14	0.355
**Females**	75 (43.86%)	19	39	17	
**Duration of surgery**	201.05 ± 43.29	178.96 ± 25.95	207.27 ± 32.68	217.90 ± 71.64	<0.001
**Total blood loss**	453.86 ± 227.99	405.90 ± 256.59	472.10 ± 218.82	476.11 ± 200.71	0.641
**Tumor type**					**<0.001**
Medulloblastoma	34	4	29	1	**<0.001**
Ependymoma	27	11	13	3	0.232
Pilocytic astrocytoma	30	13	12	5	0.137
Mixed gliomas	13	3	9	1	0.433
Choroid plexus papilloma	9	3	6	0	0.347
Craniopharyngioma	5	0	4	1	0.336
Meningeoma	4	3	0	1	0.069
Germ cell tumor	13	4	6	3	0.842
ATRT	12	2	6	4	0.314
Pineocytoma	3	1	2	0	0.712
Glioblastoma	5	2	2	1	0.815
Others	16	3	2	11	**<0.001**
**WHO grading**					0.053
I	66	21	32	13	0.615
II	19	8	5	6	**0.041**
III	11	5	4	2	0.41
IV	75	15	50	10	**0.008**
**Tumor location**					**<0.001**
Posterior fossa	88	24	57	7	**0.002**
Hemicerebrum	38	16	15	7	**0.027**
Lateral ventricle	9	2	6	1	0.698
Sellar region	22	2	7	13	**<0.001**
Pineal region	14	5	6	3	0.184
**Volume (cm^3^)**					
≤15	59	28	20	11	**0.008**
>15 and ≤35	46	8	27	35	
>35	66	13	44	9	
**Bottom vessel**					
Yes	82	19	42	21	**0.036**
No	89	30	49	10	
**Encasing nerve or pass-by vessel**					
Yes	70	20	29	21	**0.003**
No	101	29	62	10	
**Finger-like attachment**					
Yes	66	17	30	19	**0.016**
No	105	32	61	12	
**Characteristics**					
Cystic	75	32	50	16	0.292
Solid	96	17	41	15	
**Tumor depth**					
Deep	115	27	66	22	0.075
Superficial	56	22	25	9	
**Ratio of “limited line”**					
<10%	45	30	9	6	**<0.001**
≥10 and <50%	69	10	52	7	
≥50%	56	8	30	18	
**Ratio of “clear line”**					
≥90%	56	18	28	8	**0.023**
<90 and ≥50%	69	18	42	11	
<50%	46	13	21	12	
**EOR**					
Gross total	117	40	74	6	**<0.001**
Subtotal	26	9	12	5	
Partial	28	0	5	20	
**Complication rate**	75 (43.86%)	14	42	18	**0.025**
Major complications	44 (25.58%)	10	23	11	0.320
Minor complications	36 (20.93%)	5	25	6	**0.043**
Neurological complications	25 (14.53%)	3	8	14	**<0.001**

The meaning of the bold values means the results is statistically signaficant.

Multinomial logistic regression analysis showed that tumor volume, bottom vessel, encasing nerve or pass-by vessel, finger-like attachment, ratio of “limited line”, and ratio of “clear line” remained to be independent predictors for the application of the en bloc concept (**Table 3**). Compared with tumor size between 15 and 35 cm^3^, tumors with the giant size over 35 cm^3^ were more likely to be removed by en bloc concept (complete: OR = 0.154, p = 0.021; partial: OR = 0.163, p = 0.021). The lesions without finger-like attachment, bottom vessel, or encasing nerve or pass-by vessel were more likely to be removed by complete en bloc concept (OR = 6.311, p = 0.007; OR = 12.325, p = 0.001; and OR = 4.738, p = 0.016, respectively) or partial en bloc concept (OR = 7.951, p = 0.001; OR = 6.745, p = 0.002; and OR = 4.594, p = 0.007, respectively). In addition, compared with ratio of “clear line” less than 50%, the ratio over 90% (complete: OR = 10.127 p = 0.006; partial: OR = 4.862, p = 0.026) and the ratio between 50% and 90% (complete: OR = 5.826, p = 0.021; partial: OR = 4.513, p = 0.022) were both the predictors of the application of the en bloc concept in our experience. Similarly, compared with ratio of “limited line” over 50%, the ratio less than 10% (complete: OR = 35.990, p < 0.001) and the ratio between 50% and 10% (complete: OR = 9.582, p = 0.007; partial: OR = 7.720, p = 0.003) were both the predictors of the application of the en bloc concept in our medical center.

**Table 3:** Multivariate multinomial logistic regression with the piecemeal concept as the base case.

	Complete en bloc concept					Partial en bloc concept				
	Waldχ2	OR	95% CI		P-value	Waldχ2	OR	95% CI		P-value
**Volume (cm³)**										
≤15	0.4	1.622	0.363	7.256	0.527	3.63	0.267	0.069	1.039	0.057
>15 and ≤35	4.163	0.154	0.025	0.929	0.041	5.354	0.163	0.035	0.758	0.021
>35		Reference	Reference	Reference			Reference	Reference	Reference	
**Bottom vessel**										
No	13.084	12.325	3.16	48.065	<0.001	9.893	6.745	2.053	22.158	0.002
Yes		Reference	Reference	Reference			Reference	Reference	Reference	
**Encasing nerve or pass-by vessel**										
No	5.786	4.738	1.334	16.829	0.016	7.375	4.594	1.529	13.806	0.007
Yes		Reference	Reference	Reference			Reference	Reference	Reference	
**Finger-like attachment**										
Yes	7.185	6.311	1.641	24.276	0.007	10.740	7.951	2.301	27.473	0.001
No		Reference	Reference	Reference			Reference	Reference	Reference	
**Ratio of “limited line”**										
<10%	18.626	35.99	7.071	183.19	<0001	0.199	1.407	0.314	6.312	0.656
≥10 and <50%	7.379	9.582	1.876	48.932	0.007	9.12	7.72	2.049	29.083	0.003
≥50%		Reference	Reference	Reference			Reference	Reference	Reference	
**Ratio of “clear line”**										
≥90%	1.384	10.127	1.962	52.264	0.006	4.953	4.862	1.208	19.574	0.026
<90 and ≥50%	5.287	5.826	1.297	26.173	0.021	5.219	4.513	1.239	16.444	0.022
<50%		Reference	Reference	Reference			Reference	Reference	Reference	
**R2 (pseudo)**	0.572									
**No. (%) correct prediction**	70.20%									

## Discussion

In 1990, according to the infiltrating extents of lesions, Enneking and colleagues proposed an oncologic staging system concerning the primary spine tumors for the effective surgical plan including extensive resection, marginal resection, and intralesional resection (20, 21). In brain tumors, extensive resection should include excision of the lesion and removal of fluid-attenuated inversion recovery (FLAIR) abnormalities, called as “maximal resection” in some studies. The increased mortality, limited prolonged OS, different tumor spectrum, and absence of robust evidence of application in children limited the popularity of extensive resection in neurosurgeons (22–27). Hence, the complete en bloc concept in this study referred to the marginal resection as a whole excision of lesion and retention of T2-FLAIR abnormalities, considering potential dysplasia of adjacent brain parenchyma in children, resulting from the extensive resection.

As shown in **Table 1**, both the duration of resection and the blood loss of resection were decreased, indirectly contributing to the reduction of surgical time and total blood loss. As to a relative higher GTR rate, better control of intraoperative hemorrhage might decrease the flustered manipulation and indirectly increase the opportunity to perform GTR. Furthermore, the decreased blood loss and transfusion rate might ameliorate the dysfunctional cerebrospinal fluid circulation and then reduce the systemic and neurological complication rates significantly or numerically, including postoperative fever, hydrocephalus needing VPS treatment, and even perioperative mortality. Hence, it is possible and beneficial to improve the perioperative outcomes for these selective children when integrating complete or partial en bloc concept into the brain tumor surgery. Unlike the encouraging results observed in other medical divisions when applying en bloc concept, no significant difference in the recurrence rate and the metastatic rate was observed despite the numerical improvement.

As shown in **Table 2**, the “clear line” ratio of over 85% (156/171) cases was over 50%, and over 80% (146/171) had a “limited line” ratio less than 50%. As the three most common types of pediatric brain tumors, almost all of the medulloblastoma, over 80% of ependymoma and pilocytic astrocytoma were applied with en bloc concept (**Table 2**). Despite the indistinct boundary between the medulloblastoma and brain parenchyma, their texture difference could permit the marginal identification and the safe separation from normal tissue. The juvenile pilocytic astrocytoma, as one type of astrocytoma, comprises about 20% of all brain tumors (28). Generally, these tumors are cystomas with mural nodules or solid lesions with small cysts in it and are well circumscribed and noninvasive. They have relative clear boundaries with normal tissues consisting of reactive non-neoplastic tissue. Ependymomas are homogenous, extensively cystic, well-defined, and partially encapsulated lesions (29, 30). The low-grade ependymomas are cellular and have a regular histological pattern and, even for the anaplastic ependymomas, characterized by microvascular proliferation and resulting in intraoperative hemorrhage tendency. Therefore, it is possible to realize the circumferential cauterization and resection, serving as one part of en bloc concept, in the most pediatric tumors.

In addition to the inherent feature of lesions, the anatomical characteristics of different pathogenic sites also affect the application of en bloc concept. In this study, over 90% posterior fossa tumors were removed by complete or partial en bloc concept. Medulloblastoma, astrocytoma, and ependymoma account for about 70% of posterior fossa tumors of children and are all indications of the en bloc resection if surrounding structures permit (31). These lesions always grow along the path of least resistance, but most of them have expansile remolding and well-defined borders (32). Furthermore, the fixed blood source—the branches of the right superior cerebellar artery (SCA) and posterior inferior cerebellar artery (PICA)—could be a reliable reference when the circumferential feeding vessel exposure was performed. The prone position is the top choice for these lesions because of the panoramic view of posterior fossa it provides and the exposure of deep-seated lesion and identification of critical structures. If the lesions invade the brainstem, ventricular floor, cranial nerves, or other vital structures, safe debulking of these tumor tissues is advocated. As to the hemicerebrum tumor, the shallow location and craniectomy provided more operative space and better lesion exposure, so over 40% (16/38) neoplasms were removed as a whole in this study (**Table 2**). However, this method does not always work. For the lesions encasing the eloquent cortex, saving the piecemeal resection is still preferred. Because of the cystic characteristics of the lateral ventricle, exogeneity of neoplasm, and fixed feeding vessels (lenticulostriate arteries or medial posterior choroidal arteries), most tumors could be separated from the ventricular wall. The choice of surgical approach depends on the tumor location, size, origin of the arterial feeders, and the relationship between the tumor, the choroid plexus, and the internal cerebral veins. The ideal approach provides sufficient exposure of traversing and feeding vessels, the shortest trajectory to lesion, minimal injuries of functional eloquent cortex, and maximal possibility of GTR.

As mentioned before, even those cases in piecemeal resection group were also more or less applied with partial en bloc conception for better blood loss control and preservation of tumor samples, according to the intra-operative findings. Despite this, considering the hypervascular characteristics of pediatric brain tumor and small blood volume of children, the fast and safe resection rather than the surgical concept or extent of resection should be taken in consideration first, and the obstinate idea of removing as a whole or blocking the blood feeding prior to the resection should also be avoided. In addition, the giant lesions are difficult to be taken out as a whole in some cases, such as the excision of intraventricular tumor under endoscope and excision of lesions in the CPA. If possible, reshaping the tumor by bipolar coagulation after the tumor isolation is still preferred (**Figure 5B**). Compared with piecemeal resection, this method could effectively control the blood loss during the tumor resection. The tumor isolation with circumferential cauterization from the en block concept is still work in this case. Otherwise, the piecemeal resection is still the optimal choice to protect the normal brain function. We should adjust our surgical strategy to the intraoperative situation and surgeons’ experience to pursue a safe and fast surgery with the maximal resection and excellent outcomes.

### Limitations

This study still has several limitations. First, this is a single-center, retrospective study and may suffer selective bias. Second, only 56 cases were matched with a limited ability to detect the statistically significant difference of the short outcome between the piecemeal and en bloc groups, and because this was also the experience from single medical center, a large sample size was also needed to detect more predictors. Third, whether one tumor was removed by en bloc concept always depends on the intra-operative findings and the experience of surgeon. It is hard to avoid the subjective bias when including the tumor characteristics investigated in the study. However, considering the improvement of perioperative outcomes and proficient skill of Dr. Ma (J.M. with a 20-year surgical experience in adult and a 17-year surgical experience in children, member of the International Society for Pediatric Neurosurgery), this model still could be a reference for surgical plan. Fourth, according to the objective of our study, a relative short follow-up of 2 years was adopted, and a robust illustration of the effect on the recurrent rate, metastatic rate, and other long-term efficiencies was deficient. Therefore, further prospective stratified control study with enough sample size and long follow-up is needed, and perioperative quantitative determination of exfoliated cancer cells in blood or cerebrospinal fluid is also deserved in the future study.

## Conclusion

This study supports the application of complete or partial en bloc concept in the pediatric brain tumor surgery referring to the preoperative imaging features, and compared with piecemeal concept, the en bloc concept can improve the short outcomes without significant increase in neurological complication.

## Data availability statement

The original contributions presented in the study are included in the article/**Supplementary Material**. Further inquiries can be directed to the corresponding authors.

## Ethics statement

Ethical review and approval was not required for the study on human participants in accordance with the local legislation and institutional requirements. Written informed consent to participate in this study was provided by the participants’ legal guardian/next of kin.

## Author contributions

LC: Methodology (lead) and writing —original draft (lead). ST: Data collection (equal), methodology (equal), writing— original draft (lead), and writing—review and editing (equal). WM: Data collection (equal). Writing— review and editing (lead). ZN: Data collection (equal). GT: Statistical methodology (equal). YZ: Data collection and Funding acquisition (equal). BW: Writing—review and editing (equal). QW: Data collection and writing—review and editing (equal). ZX: Data collection (equal). JW: Methodology (equal) and writing —review and editing (equal). ZL: Data collection (equal). HZ: Data collection (equal). LY: Data collection (equal). JM: Study design, performing surgery, writing—review and editing (equal), and funding acquisition (equal). All authors contributed to the article and approved the submitted version.

## Funding

This research was supported by the Shanghai Leading Talent Program (to JM), the National Science Foundation of China for Young Scholars (No. 81702453 to YZ), Shanghai Science and Technology Committee (No. 17411965700 to YZ and No. 17411951800 and No. 19411952100 to JM), Joint Research of Medicine and Industry of Shanghai Jiao Tong University (No. YG2015QN42 to YZ), Shanghai Xin Hua Hospital (No. JZPI201701 to JM), and Shanghai Shen Kang Hospital Development Center (No. 16CR2031B to JM).

## Conflict of interest

The authors declare that the research was conducted in the absence of any commercial or financial relationships that could be construed as a potential conflict of interest.

## Publisher’s note

All claims expressed in this article are solely those of the authors and do not necessarily represent those of their affiliated organizations, or those of the publisher, the editors and the reviewers. Any product that may be evaluated in this article, or claim that may be made by its manufacturer, is not guaranteed or endorsed by the publisher.
