# Supplementary figures and images for: The tentative application of en bloc concept in the pediatric brain tumor: Experience from a large pediatric center in china
Source: Front Oncol. 2022 Nov 11;12:1018380. doi: 10.3389/fonc.2022.1018380 (PMC9697186; doi:10.3389/fonc.2022.1018380)

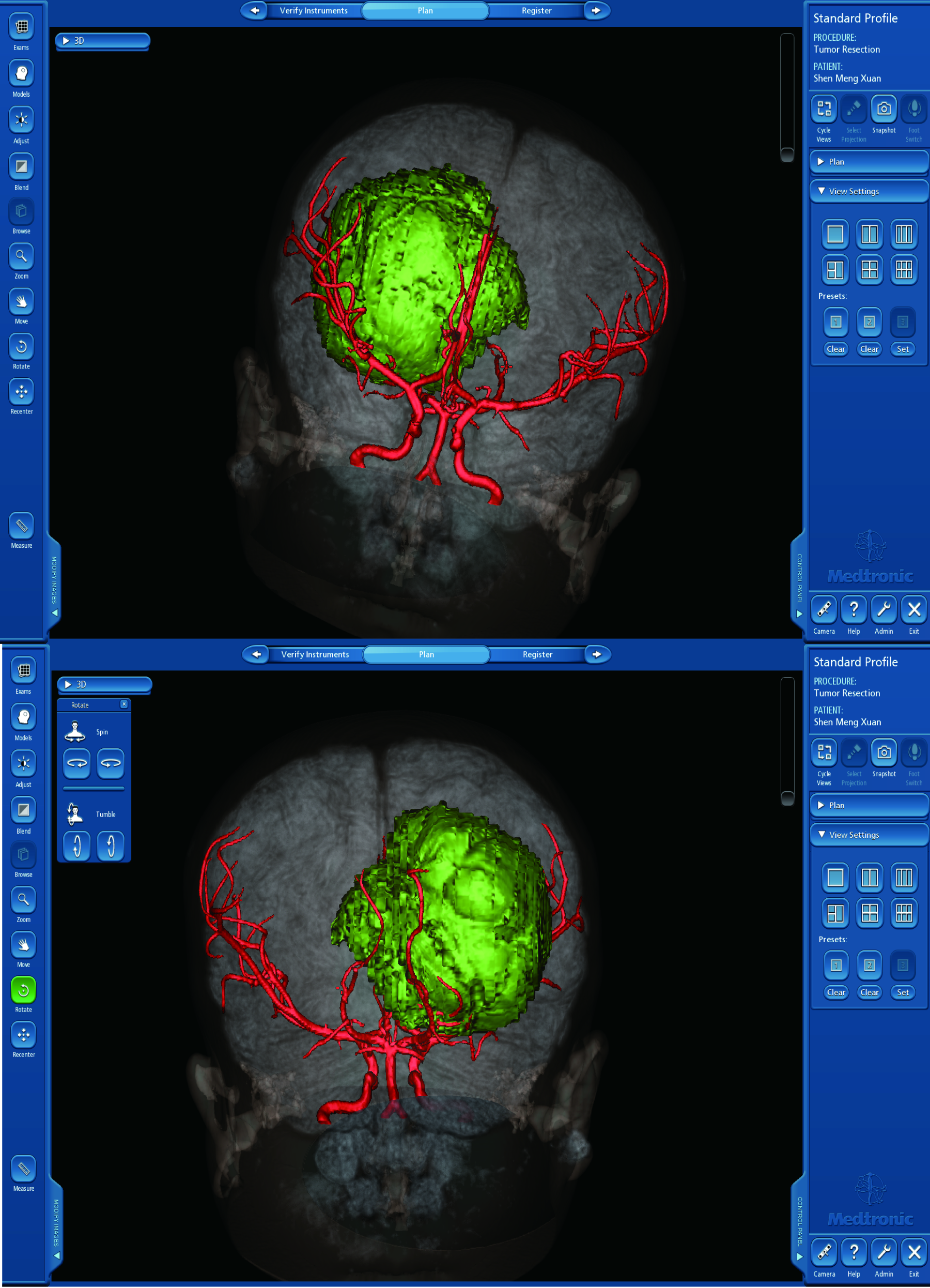

Supplement: Supplementary file 1 [file Image_1.tif]

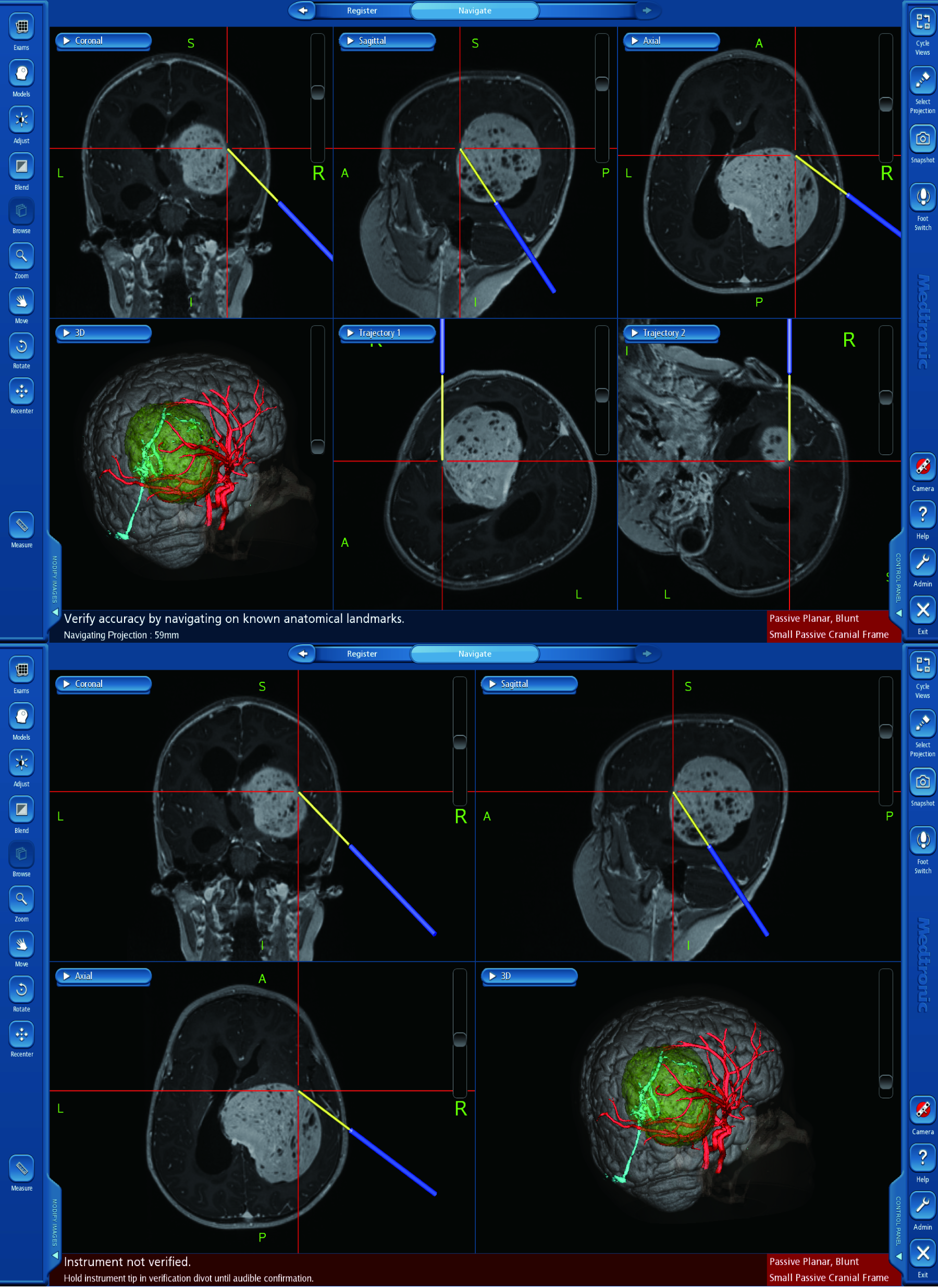

Supplement: Supplementary file 2 [file Image_2.tif]

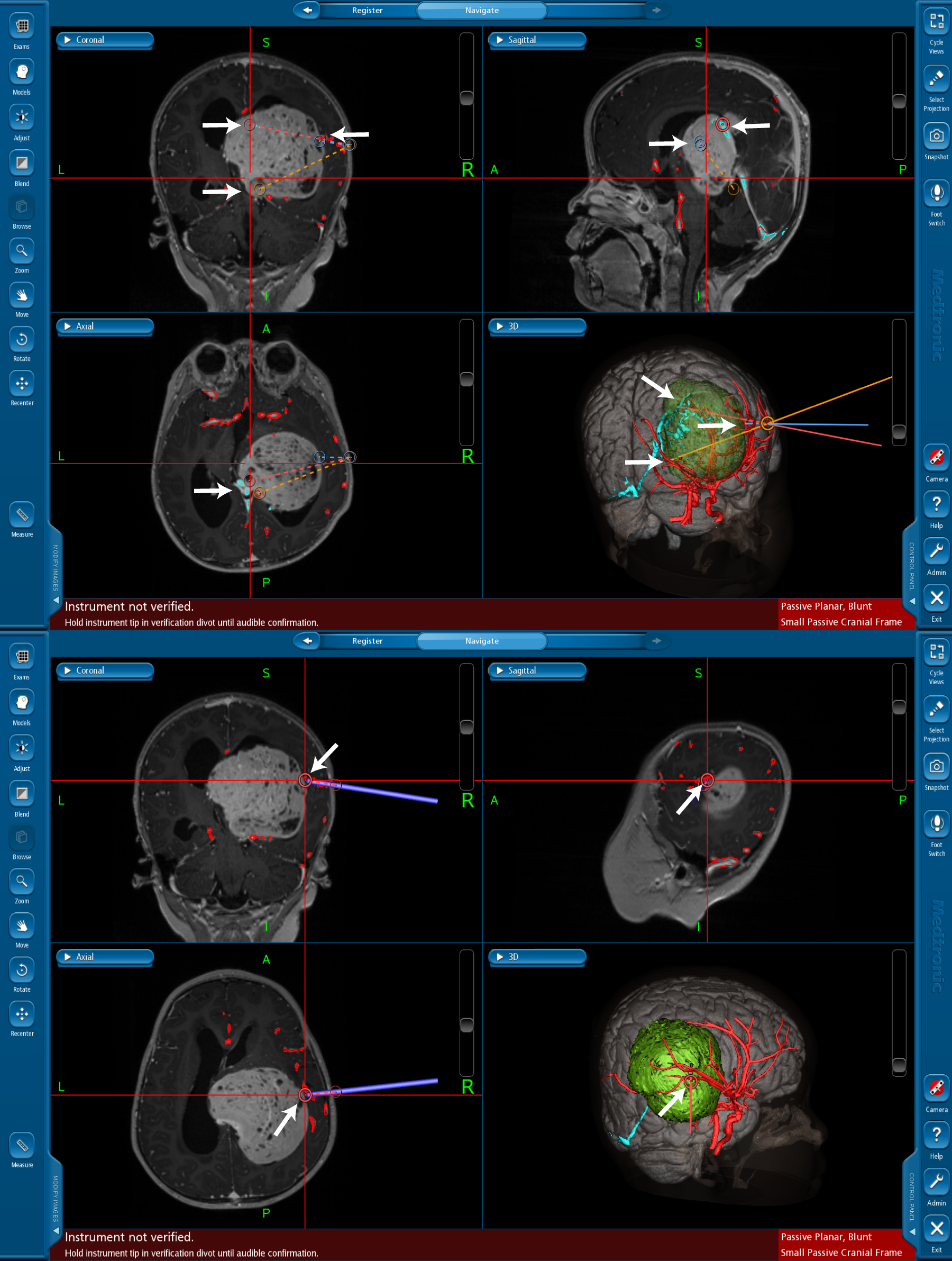

Supplement: Supplementary file 3 [file Image_3.tif]
